# Comparing thousands of circular genomes using the CGView Comparison Tool

**DOI:** 10.1186/1471-2164-13-202

**Published:** 2012-05-23

**Authors:** Jason R Grant, Adriano S Arantes, Paul Stothard

**Affiliations:** 1 Department of Agricultural, Food and Nutritional Science, University of Alberta, Edmonton, AB, T6G2P5, Canada

## Abstract

****Background**:**

Continued sequencing efforts coupled with advances in sequencing technology will lead to the completion of a vast number of small genomes. Whole-genome comparisons represent an important part of the analysis of any new genome sequence, as they can provide a better understanding of the biology and evolution of the source organism. Visualization of the results is important, as it allows information from a variety of sources to be integrated and interpreted. However, existing graphical comparison tools lack features needed for efficiently comparing a new genome to hundreds or thousands of existing sequences. Moreover, existing tools are limited in terms of the types of comparisons that can be performed, the extent to which the output can be customized, and the ease with which the entire process can be automated.

****Results**:**

The CGView Comparison Tool (CCT) is a package for visually comparing bacterial, plasmid, chloroplast, or mitochondrial sequences of interest to existing genomes or sequence collections. The comparisons are conducted using BLAST, and the BLAST results are presented in the form of graphical maps that can also show sequence features, gene and protein names, COG (Clusters of Orthologous Groups of proteins) category assignments, and sequence composition characteristics. CCT can generate maps in a variety of sizes, including 400 Megapixel maps suitable for posters. Comparisons can be conducted within a particular species or genus, or all available genomes can be used. The entire map creation process, from downloading sequences to redrawing zoomed maps, can be completed easily using scripts included with the CCT. User-defined features or analysis results can be included on maps, and maps can be extensively customized. To simplify program setup, a CCT virtual machine that includes all dependencies preinstalled is available. Detailed tutorials illustrating the use of CCT are included with the CCT documentation.

****Conclusion**:**

CCT can be used to visually compare a reference sequence to thousands of existing genomes or sequence collections (next-generation sequencing reads for example) on a standard desktop computer. It provides analysis and visualization functionality not available in any existing circular genome visualization tool. By visually presenting sequence conservation information along with functional classifications and sequence composition characteristics, CCT can be a useful tool for identifying rapidly evolving or novel sequences, horizontally transferred sequences, or unusual functional properties in newly sequenced genomes. CCT is freely available for download at http://stothard.afns.ualberta.ca/downloads/CCT/.

## Background

The analysis of a newly sequenced bacterial genome often involves comparing the sequence to previously characterized genomes. Such comparisons can help to identify novel or rapidly evolving sequences, horizontal sequence transfer events, and interesting functional differences or relationships. Tools that can be used to visualize sequence conservation in conjunction with other sequence characteristics, such as functional classifications and nucleotide composition, are particularly popular [1-3]. Continual advances in sequencing technology have contributed to the availability of numerous bacterial genome sequences—currently there are thousands of bacterial and archaeal genome sequences available, and undoubtedly many more on the way. Given the accessibility of genome sequences there is an increasing need for comparative genomics tools that can handle large numbers of sequences. The CGView Comparison Tool (CCT) is a software package designed for visually comparing bacterial, plasmid, chloroplast, or mitochondrial genomes to thousands of other genomes or sequence collections. CCT can also display sequence feature information, COG classifications (which it determines itself), sequence analysis results, and base composition plots. Maps are highly customizable, both in terms of how comparisons are conducted and how results are presented, and can be generated in several sizes and file formats, suitable for publications, presentations, and posters.

## Implementation

CCT consists of several custom Bash and Perl scripts that are used to create a local COG sequence database [4], download sequences of interest from NCBI [5], extract and translate ORFs, extract CDS feature translations, perform BLAST searches [6], assign COG functional categories to proteins, generate CGView XML files [7] and create graphical maps. Additional scripts are included for redrawing maps and for drawing zoomed maps showing regions of interest in more detail. Wrapper scripts simplify the use of CCT by allowing a single command to run many of the CCT components in succession. The usage of these scripts is described in the “commands” section of the CCT documentation and demonstrated in the CCT tutorials. Although a command-line interface can be intimidating, the availability of a Linux virtual machine and clear documentation and tutorials should help users with little command-line experience take advantage of CCT. Furthermore, a few simple commands can be used to create several complex maps, thanks to the included wrapper scripts. Command-line tools are widespread and popular in bioinformatics, and offer important advantages related to reproducibility, automation, and remote execution.

## Results

### **Creating maps using CCT**

CCT maps typically consist of several rings depicting a reference genome and its features (coding sequences and ORFs for example), and the results of BLAST comparisons between the reference sequence and one or more comparison sequences. A separate BLAST ring is drawn for each comparison genome. When there is similarity between a portion of the reference sequence and any part of a comparison sequence, a coloured arc is drawn beneath the region of the reference sequence showing similarity, in the appropriate comparison ring. User defined features, analysis results, and base composition plots may also be displayed.

**Figure 1:**
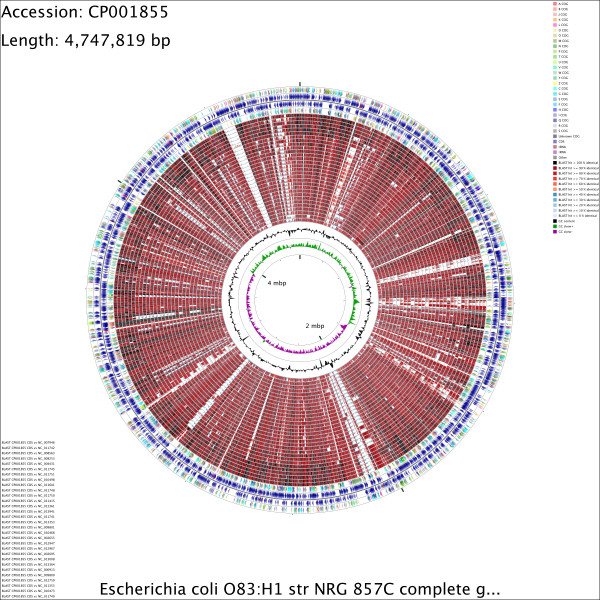
**CCT map comparing an *E. coli *reference sequence to other E. coli genomes.** This map was generated by commands 1 to 5 given in Additional file 1A. Starting from the outermost ring the feature rings depict: 1. COG functional categories for forward strand coding sequences; 2. Forward strand sequence features; 3. Reverse strand sequence features; 4. COG functional categories for reverse strand coding sequences. The next 30 rings show regions of sequence similarity detected by BLAST comparisons conducted between CDS translations from the reference genome and 30 *E. coli* comparison genomes. The last two rings display the GC content and GC skew.

**Figure 2:**
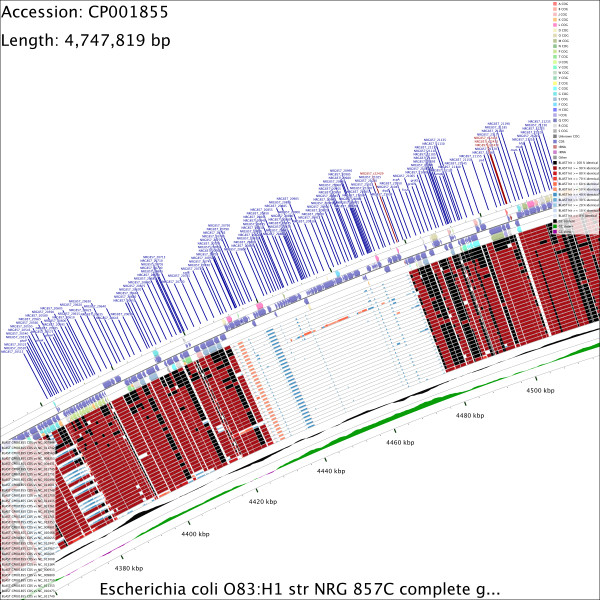
**Zoomed CCT map showing enhanced detail.** This 15X zoomed map was produced by command 6 given in Additional file 1A. Starting from the uppermost slot the feature slots depict: 1. COG functional categories for forward strand coding sequences; 2. Forward strand sequence features; 3. Reverse strand sequence features; 4. COG functional categories for reverse strand coding sequences. The next 30 slots show regions of sequence similarity detected by BLAST comparisons conducted between CDS translations from the reference genome and 30 *E. coli* comparison genomes. The last two slots show the GC content and GC skew.

There are three main approaches to drawing maps using CCT. One is to run the *cgview_comparison_tool.pl* script, supplying it with the name of a new map project (Additional file 1A). The script creates a directory tree named after the project, consisting of a few directories and a configuration file (Additional file 1B). The user then places the sequence to be drawn and the comparison sequences into the reference_genome and comparison_genomes directories, respectively. Several sequence formats are supported, including GenBank, EMBL, and FASTA. Protein or DNA sequence collections in FASTA format (sequence reads for example) can also be placed in the comparison_genomes directory. Additional features or analysis results (gene expression measurements for example) can be placed in the appropriate directories at this time. The configuration file, which is a simple text file consisting of options and values as well as explanations of the options can be edited to specify, for example, the types of BLAST comparisons to be performed, the base composition plots to display, and the size of the map to be generated. When the *cgview_comparison_tool.pl* script is rerun on the same project, it detects the sequence files and initiates the map creation process. All intermediate and output files, including the BLAST results, COG assignments, CGView XML files, and graphical maps, are written to directories located inside the project directory. A sample map generated using this script is given in Figure 1. Once the map has been completed the *create_zoomed_maps.sh* and *redraw_maps.sh* scripts can be used to draw additional maps showing regions of interest in more detail (Figure 2), or to make changes to the appearance of the map. Both scripts make use of the existing BLAST results in the project directory so that the BLAST searches are not repeated.

**Figure 3:**
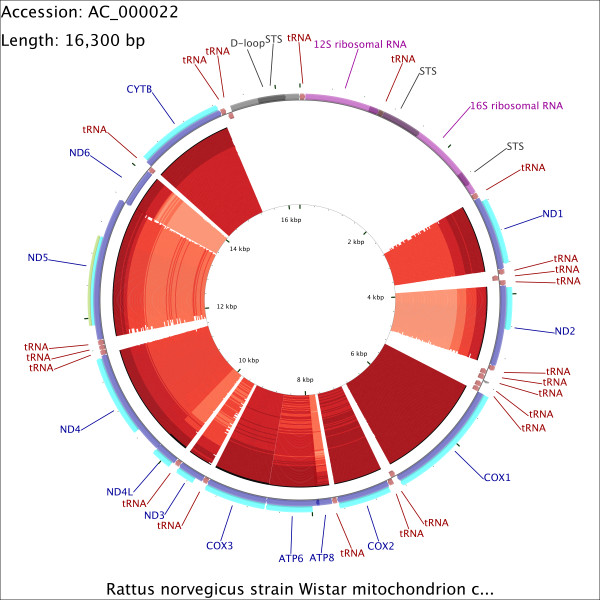
**CCT map comparing the rat mitochondrial genome to itself and 99 other mitochondrial genomes.** This map was generated by commands given in Additional file 2A. Starting from the outermost ring the feature rings depict: 1. COG functional categories for forward strand coding sequences; 2. Forward strand sequence features; 3. Reverse strand sequence features; 4. COG functional categories for reverse strand coding sequences (the ring is empty because no COG categories were assigned). The remaining rings show regions of sequence similarity detected by BLAST comparisons conducted between CDS translations from the reference genome and 100 comparison genomes.

The second and even simpler approach to using CCT is to run the *build_blast_atlas.sh* script (Additional file 2), which accepts a GenBank file as input and creates several graphical maps comparing the input sequence to all sequences placed in the comparison_genomes directory. The advantage of using this wrapper script is that it automatically creates two map projects, one comparing the sequences at the DNA level using blastn searches, and one comparing the CDS feature translations using blastp. The two map types are useful for different purposes: conserved non-transcribed or non-translated sequences can be identified using the blastn map, while similar protein coding regions are best visualized using the blastp map. The CDS maps also display COG functional categories, which are assigned automatically by CCT (Figure 3). Both map types also use a modified colour scheme for BLAST hits (hits are coloured based on sequence identity as opposed to source genome). For each map type three images are generated showing differing levels of detail. The largest map includes feature labels for the reference genome. Expanded maps showing more detail can be created using the *create_zoomed_maps.sh* script. Examples of larger maps comparing thousands of genomes are available on the CCT web site.

**Figure 4:**
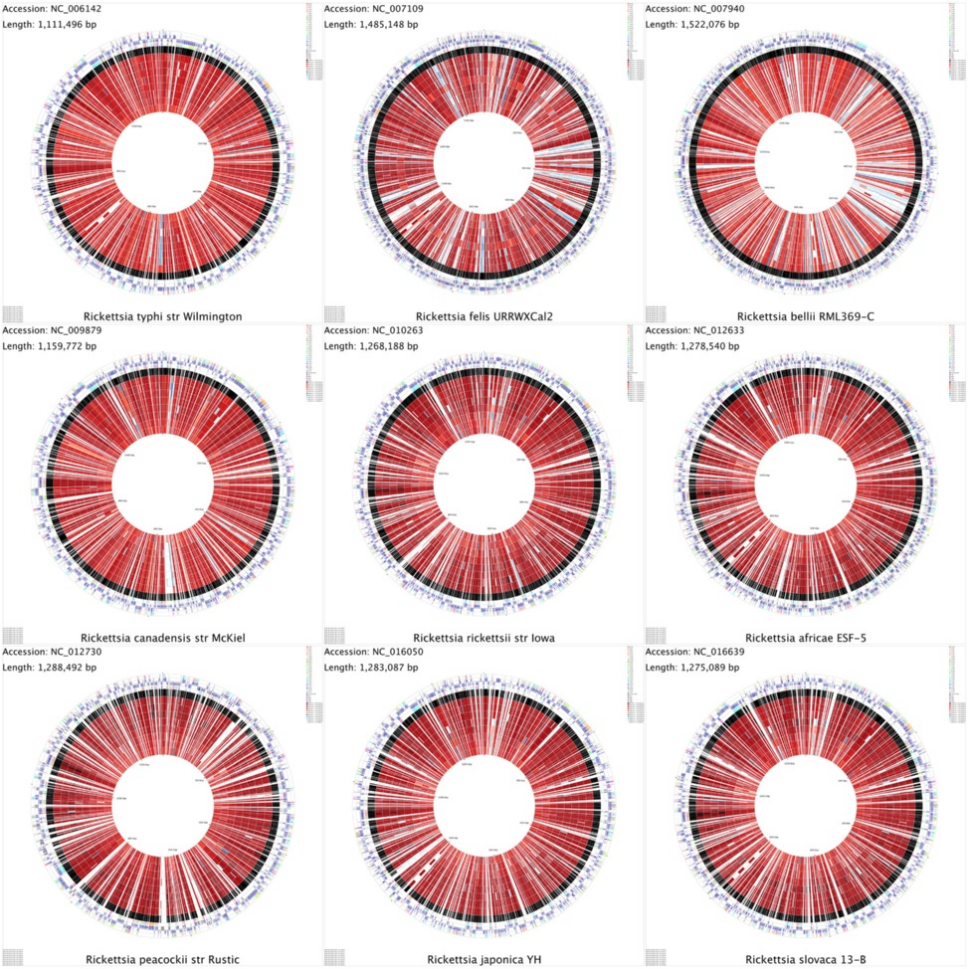
**Montage of CCT maps comparing nine *Rickettsia *genomes.** A montage of maps comparing *Rickettsia* genomes at the protein level, created using the *build_blast_atlas_all_vs_all.sh* script. The montage is comprised of nine separate CCT maps, each depicting one of the genomes as the reference and the remaining eight as the comparison genomes. Starting from the outermost ring the four features rings depict: 1. COG functional categories for forward strand coding sequences; 2. Forward strand sequence features; 3. Reverse strand sequence features; 4. COG functional categories for reverse strand coding sequences. The remaining rings show regions of similarity detected using BLAST (blastp).

The third approach to using CCT is to run the *build_blast_atlas_all_vs_all.sh* script. This script creates a separate map for every sequence in the comparison_genomes directory, depicting the results of BLAST comparisons with all the other sequences in the directory. The individual maps are then combined into a single montage map (Figure 4). The montage allows more thorough comparisons to be conducted among a group of closely related genomes, by allowing every sequence to serve as the reference sequence. For example, sequences missing from a reference genome relative to the comparison genomes will not be observed using a single map but can be detected using this approach. The separate (and larger) map images combined to create the montage are also available in the output directory.

An important and potentially time-consuming step when creating maps, regardless of the mapping software used, is obtaining the reference and comparison genome sequence records to be visualized. To simplify this process CCT includes several scripts for downloading sequences of interest directly from NCBI. These scripts make use of the NCBI Entrez Utilities Web Service API and can be used to download a single record based on accession number (*fetch_genome_by_accession.sh*), all the records corresponding to a particular species or genus (*fetch_refseq_bacterial_genomes_by_name.sh*), or all the records from a particular organelle or domain (e.g. *fetch_all_refseq_mitochondrial_genomes.sh*). Options are included for further restricting sequence retrieval according to sequence length. These options allow, for example, plasmid sequences to be avoided when downloading bacterial chromosomes. As with the other CCT scripts, the use of these utilities is demonstrated in the tutorials and all script options are described in the “commands” section of the CCT documentation.

### **CCT visualization enhancements**

Several subtle but important enhancements have been made to CCT during its development to increase its utility as a genome visualization tool. For example, features parsed from reference sequence file are drawn with partial opacity by default, so that the boundaries of features are apparent even if they overlap. When CCT assigns COG functional categories, it is able to appropriately handle cases where a protein appears to fall into multiple categories (based on sequence comparisons). In such situations the coloured arrow used to indicate COG class is divided into the appropriate number of smaller arrows, each coloured to represent a single COG category. This behaviour differs from that used to create virtually all existing bacterial maps that colour proteins according to functional class. When BLAST atlases are built by CCT (using the *build_blast_atlas.sh* or *build_blast_atlas_all_vs_all.sh* scripts), the reference genome is included automatically as one of the comparison genomes. The inclusion of the reference leads to a seemingly superfluous reference vs. reference BLAST analysis that actually serves an important purpose—it reveals portions of the reference that are unable to produce BLAST hits, due to ambiguous bases, BLAST filtering, or an absence of protein-coding sequences (in the case of protein or translated BLAST searches). Without the reference vs. reference ring there is the potential for comparison rings to be interpreted incorrectly. Unlike the other BLAST atlas tools we have encountered, CCT adjusts the order of the comparison genome rings automatically (when the *build_blast_atlas.sh* or *build_blast_atlas_all_vs_all.sh* scripts are used), so that the most similar genomes tend to be placed closest to the reference sequence ring. Similarity is determined using a heuristic that considers the total number of comparison genome bases contributing to hits as well as the scores of the hits. In practice this sorting makes it much easier to visualize sequence divergence trends for the genes or proteins in a reference sequence. For example, the most labile or divergent portions of a reference genome standout as light-coloured regions adjacent to the reference ring, while well-conserved portions of the reference give rise to darkly-coloured arcs that form “spikes” of conservation extending towards the centre of the map. This sorting can also reveal genome segments whose similarity is inconsistent with the general trends revealed by the map. For example, a horizontally transferred segment can appear as a dark red or black arc in a portion of the map consisting of otherwise weakly similar genomes. Multiple regions of a comparison genome can be similar to a given region of the reference genome. For this reason CCT arranges the arcs within each BLAST ring so that low-similarity arcs do not obscure higher-similarity arcs. Finally, CCT has the ability to divide protein-search BLAST rings into six sub-rings corresponding to the six different reading frames of the reference sequence. This feature, when used with zoomed maps and CCT’s ORF drawing option, can be used to distinguish which of the ORFs or CDS features in an overlapping set are conserved.

### **BLAST comparison options**

CCT writes a configuration file to each project when it is initialized. This simple file can be edited to specify, among other things, which types of BLAST comparisons are performed. In total there are currently 16 comparison scenarios available. These are described in detail in the CCT documentation, and differ primarily in terms of which regions of the sequences are compared and whether the comparisons are done at the DNA or protein level. For example, CCT can compare the entire reference sequence to each comparison genome at the nucleotide level using blastn, or it can conduct the comparisons using the 6-frame translations of the sequences, using tblastx. Alternatively, CCT can identify and translate ORFs in each sequence, and compare the translations using blastp. Instead of determining ORFs, CCT can simply extract the CDS feature translations from the input files and compare them using blastp. Finally, instead of single genomes the comparison sequence files can consist of multiple DNA sequences in FASTA format (next-generation sequencing reads for example) or multiple protein sequences in FASTA format (a custom collection of bacteriophage proteins for example). There are options specific to such multi-FASTA files for controlling how the comparisons with the reference are performed. CCT uses file extensions to determine which sequence files should be used for a given BLAST comparison. When there are multiple files with the same extension, a separate BLAST comparison is conducted for each, and the results are shown in separate rings on the resulting map. Multiple comparison types can be shown on a single map. In this manner conserved non-translated sequences can be visualized along with conserved coding regions.

### **Adding custom features and analysis results to CCT maps**

There are numerous data types users may wish to visualize on CCT maps, such as gene expression measurements, the positions of horizontally transferred segments revealed by specialized programs, the positions of SNPs identified by sequencing, and so on. To accommodate user-supplied information, all CCT projects include a “features” and an “analysis” directory. Simple tab-delimited or comma-delimited text files can be added to these directories. Files describing the positions of genes and other regions of interest are generally placed in the features directory, whereas files assigning numerical scores (positive or negative) to genome regions are placed in the analysis directory. CCT parses the files and creates a separate ring for each on the final map. The regions obtained from the features directory are drawn much like the features extracted from GenBank files, whereas those parsed from analysis files are scaled according to the accompanying score value.

### **Adjusting the appearance of CCT maps**

The maps generated by CCT are designed to be visually appealing and informative. Nonetheless the default colours, font sizes, line widths, etc. may not always be appropriate. CCT includes functionality that allows maps to be customized without the need to repeat the computationally intensive analysis steps used to assign COG categories and identify sequence similarities. This functionality is implemented using more than 70 "customization keys", which can be passed to certain CCT commands along with their desired values. For example, the "backgroundColor = rgb(0,0,0)" key-value pair can be supplied to the *build_blast_atlas.sh* script, to specify that maps should be drawn with a black background. The script can reuse existing BLAST results if they are available in the relevant project directories (using the *--start_at_xml* option). A comprehensive list of the CCT customization keys is available on the CCT web site, and commands that make use of these keys are included in the CCT tutorials.

The CCT map creation process generates an XML file for input to the CGView program [7]. This intermediate XML file, which uses a simple syntax to fully describe the features and appearance of the map, can be edited as another way of adjusting CCT maps. The *redraw_maps.sh* script can be used to quickly redraw maps directly from the modified XML file.

A final option for customization is to edit the resulting images themselves. CCT maps are drawn in PNG format by default but can instead be saved in a vector-based format (SVG). Use of SVG format allows map elements to be individually adjusted using a vector graphics editor.

## Discussion

There are many visual comparative genomics tools available, each with different advantages and disadvantages. For bacterial genomes, we previously created the CGView Server [8], which can compare a reference genome to up to three comparison genomes, using nucleotide or translated nucleotide BLAST searches. CCT greatly exceeds the capabilities of this server in all regards. For example, far more genomes can be compared, comparisons can be conducted at the level of CDS features or ORFs, much larger maps can be drawn, maps can be saved in vector or raster-based formats, COG functional categories can be assigned and shown, map appearance (colours, font sizes, feature widths etc.) can be customized extensively, comparison genome sets can be prepared much more easily, subsets of genes can be labeled, and multiple maps can be generated automatically for a set of sequences. Users also have access to the raw BLAST results, CGView XML files, and program source code. Depending on the reasons for performing the sequence comparison, other tools may be preferred. For example, ACT [1] and Circos [9] use lines to connect regions of sequence similarity between sequence ideograms, and can thus be used to visualize sequence rearrangements. Some users may prefer the default colour scheme and appearance of maps produced using the BLASTAtlas service [3], over those generated by CCT. The MultiPipmaker server can compare larger genomes and create linear figures [2]. The BLAST Ring Image Generator (BRIG) [10] uses CGView [7] to render maps and is operated using a graphical user interface. The graphical interface and included functionality for draft genomes and next-generation sequencing data files make BRIG an excellent alternative to CCT for some users and data sets. It is worth noting however that CCT has advantages over all of these tools related to its visual enhancements, diversity of included analyses, the number of genomes it can handle, its integrated scripts for downloading genomes, support for map customization, and its command-line interface for rapid creation of complex maps and pipeline integration.

## Conclusion

In summary, the CGView Comparison Tool (CCT) is a package for visually comparing circular sequences of interest to existing genomes or sequence collections. The capacity and capabilities of CCT distinguish it from existing sequence visualization tools. The ease with which large and complex maps can be created should make CCT appealing to anyone who aims to learn more about bacterial, plasmid, chloroplast, or mitochondrial genome sequences.

## Availability and requirements

**Project name:** CGView Comparison Tool

**Project home Page:**http://stothard.afns.ualberta.ca/downloads/CCT/

**Operating system(s):** Unix/Linux

**Programming language:** Perl **License:** GNU GPL

**Any restrictions to use by non-academics:** none

## Competing interests

The authors declare that they have no competing interests.

## Authors’ contributions

PS drafted the manuscript with assistance from JRG. PS and JRG wrote the CCT software and documentation. ASA developed the CCT virtual machine, wrote documentation, and assisted with program testing. All authors read and approved the final manuscript.

## Supplementary Material

Additional file 1 **Example CCT workflow using the *cgview_comparison_tool.pl *script.** (A) Commands for comparing a bacterial genome of interest, *E. coli* NRG857c (O83:H1), to all other *E. coli* genomes available in NCBI’s RefSeq collection. Note that these commands complete the entire map creation process, from downloading the sequence files to generating a regular and zoomed map in PNG format. (B) The directory structure of the CCT project created using the *cgview_comparison_tool.pl* command. The bold items are directories and the regular items are files. Click here for file

Additional file 2 **Example CCT workflow using the *build_blast_atlas.sh *script.** A) Commands to create a BLAST atlas comparing the *Rattus norvegicus* mitochondrial genome to all mitochondrial genomes available in NCBI’s RefSeq collection. Note that by default the 100 most similar comparison genomes are selected by CCT after BLAST analysis for inclusion on the final map. B) The directory structure created by the *build_blast_atlas.sh* command. The bold items are directories and the regular items are files. Click here for file
