# Supplementary material for: Comparing thousands of circular genomes using the CGView Comparison Tool
Source: BMC Genomics. 2012 May 23;13:202. doi: 10.1186/1471-2164-13-202 (PMC3469350; doi:10.1186/1471-2164-13-202)
Supplement: Additional file 1 — Example CCT workflow using the cgview_comparison_tool.pl script. (A) Commands for comparing a bacterial genome of interest, E. coli NRG857c (O83:H1), to all other E. coli genomes available in NCBI’s RefSeq collection. Note that these commands complete the entire map creation process, from downloading the sequence files to generating a regular and zoomed map in PNG format. (B) The directory structure of the CCT project created using the cgview_comparison_tool.pl command. The bold items are directories and the regular items are files. [file 1471-2164-13-202-S1.pdf]

A - Example Project Flow using *cgview_comparison_tool.pl*

1) Create a new project called `my_project` with the directory structure seen in **B**:

```
[CCT]$ cgview_comparison_tool.pl -p my_project
```

2) Download the NRG857c chromosome sequence (AC: CP001855) into the `reference_genome` directory:

```
[CCT]$ fetch_genome_by_accession.sh -a CP001855 -o my_project/reference_genome/
```

3) Download all complete *E. coli* genome sequences in GenBank into the `comparison_genomes` directory:

```
[CCT]$ fetch_refseq_bacterial_genomes_by_name.sh -n "Escherichia*" \
       --min 1000000 -o my_project/comparison_genomes/
```

`--min`: restrict sequences returned to greater than 1 MB in length to remove plasmids from the analysis.

4) Edit the `project_settings.conf` file to control how CCT processes the project. For example, to perform a blast with CDS vs CDS and view COG functional categories, find and change the following three lines:

reference genome	comparison genomes	reference COGs
query_source = none	*database_source = none*	*cog_source = none*
↓	↓	↓
query_source = cds	*database_source = cds*	*cog_source = cds*

5) Run CCT again to generate the comparison map (shown in Figure 1) as a PNG image in the `maps` directory:

```
[CCT]$ cgview_comparison_tool.pl -p my_project --cct -t
```

`--cct`: causes BLAST rings to be more prominent and coloured according to the percent identity of each hit.
`-t`: sort BLAST results such that genomes with highest similarity are plotted first.

6) Additional maps can be drawn to zoom in on an area and change the file format (map shown in Figure 2):

```
[CCT]$ create_zoomed_maps.sh -p my_project -c 4450000 -z 15 -f svg
```

`-c`: nucleotide position to centre map on
`-z`: zoom multiplier
`-f`: map image type

B - CCT Project Directory Structure

```
my_project/
├── reference_genome        - directory to place reference genome
├── comparison_genomes      - directory to place multiple comparison genomes
├── analysis                - directory to place optional analysis GFF files
├── features                - directory to place optional feature GFF files
├── blast                   - directory where blast results are created
├── maps                    - directory that will contain final maps
├── project_settings.conf   - customize maps by editing this configuration file
├── cgview_xml_builder.pl   - script that generates the XML files used to create maps
└── log.txt                 - log of project progress
```
