# Supplementary material for: Comparing thousands of circular genomes using the CGView Comparison Tool
Source: BMC Genomics. 2012 May 23;13:202. doi: 10.1186/1471-2164-13-202 (PMC3469350; doi:10.1186/1471-2164-13-202)
Supplement: Additional file 2 — Example CCT workflow using the build_blast_atlas.sh script. A) Commands to create a BLAST atlas comparing the Rattus norvegicus mitochondrial genome to all mitochondrial genomes available in NCBI’s RefSeq collection. Note that by default the 100 most similar comparison genomes are selected by CCT after BLAST analysis for inclusion on the final map. B) The directory structure created by the build_blast_atlas.sh command. The bold items are directories and the regular items are files. [file 1471-2164-13-202-S2.pdf]

A - Example Project Flow using *build_blast_atlas.sh*

1) Create a new project called `my_project` with the directory structure seen in **B**:

```
[CCT]$ build_blast_atlas.sh -p my_project
```

2) Download the *R. norvegicus* mitochondrial sequence (AC: AC_000022) into the `reference_genome` directory:

```
[CCT]$ fetch_genome_by_accession.sh -a AC_000022 -o my_project/reference_genome
```

3) Download all mitochondrial genome sequences in GenBank into the `comparison_genomes` directory:

```
[CCT]$ fetch_all_refseq_mitochondrial_genomes.sh -o my_project/comparison_genomes
```

4) Run *build_blast_atlas.sh* again to generate maps of both nucleotide (blastn) comparisons and translated coding sequence (blastp) comparisons (shown in Figure 3):

```
[CCT]$ build_blast_atlas.sh -p my_project \
       --custom "global_label=T labelFontSize=50 draw_divider_rings=F \
                 legend=F featureSlotSpacing=0.1"
```

`--custom`: settings used to customize the appearance of the map.

B - BLAST Atlas Project Directory Structure

```
my_project/
├── reference_genome                - directory to place reference genome
├── comparison_genomes              - directory to place multiple comparison genomes
├── maps_for_cds_vs_cds             - directory for final CDS maps
├── maps_for_dna_vs_dna             - directory for final DNA maps
├── analysis                        - directory to place optional analysis GFF files
├── features                        - directory to place optional feature GFF files
├── cct_projects                    - directory of CCT sub projects for DNA and CDS
│   ├── cds_vs_cds                  - CDS CCT sub project
│   └── dna_vs_dna                  - DNA CCT sub project
├── project_settings_cds_vs_cds.conf  - alter to modify CDS project
└── project_settings_dna_vs_dna.conf  - alter to modify DNA project
```
